# Conflict Adaptation and Cue Competition during Learning in an Eriksen Flanker Task

**DOI:** 10.1371/journal.pone.0167119

**Published:** 2016-12-12

**Authors:** Rodica Ghinescu, Todd R. Schachtman, Ashley K. Ramsey, Gabriele Gratton, Monica Fabiani

**Affiliations:** 1 Department of Social and Behavior Sciences, Lincoln University, Jefferson City, Missouri, United States of America; 2 Department of Psychological Sciences, University of Missouri, Columbia, Missouri, United States of America; 3 Department of Psychology, Truman State University, Kirksville, Missouri, United States of America; 4 Department of Psychology, University of Illinois at Urbana-Champaign, Champaign, Illinois, United States of America; Vrije Universiteit Brussel, BELGIUM

## Abstract

Two experiments investigated competition between cues that predicted the correct target response to a target stimulus in a response conflict procedure using a flanker task. Subjects received trials with five-character arrays with a central target character and distractor flanker characters that matched (compatible) or did not match (incompatible) the central target. Subjects’ expectancies for compatible and incompatible trials were manipulated by presenting pre-trial cues that signaled the occurrence of compatible or incompatible trials. On some trials, a single cue predicted the target stimulus and the required target response. On other trials, a second redundant, predictive cue was also present on such trials. The results showed an effect of competition between cues for control over strategic responding to the target stimuli, a finding that is predicted by associative learning theories. The finding of competition between pre-trial cues that predict incompatible trials, but not cues that predict compatible trials, suggests that different strategic processes may occur during adaptation to conflict when different kinds of trials are expected.

## Introduction

Humans are capable of providing (or withholding) responses to stimuli when such a behavior is required (or not)–revealing their ability to control their attention and action towards these stimuli. For instance, we can press the pedal to accelerate a vehicle when a traffic light becomes green, but only if the intersection is clear. It can be logically argued that this ability, that we can label “cognitive control,” must involve using contextual cues to activate (or inhibit) specific perception-action processing routes (hence the notion of “prepared reflex”, [1]). It can be hypothesized that cognitive control is used by subjects to improve their performance (providing quicker and/or more accurate behavior), based on expectations about upcoming conditions stemming from the use of contextual cues. Three questions involving cognitive control are addressed by the present study. First, what are the rules that regulate the formation of these expectations? Do they follow the rules of associative learning? Second, are these expectancies based on explicit or implicit knowledge of the cues? Finally, does latent associative learning necessarily occur during such conditions, or can the occurrence of such learning be reduced or prevented in case of redundancy between contextual cues? These issues are discussed below.

To address these questions an experimental task is needed to allow assessment of the occurrence of cognitive control, and relate it directly to contextual cues. One such task that has recently gained a lot of attention is the conflict adaptation paradigm (see [2]). This task is a conflict task: reaction times are typically slower (and responses less accurate) to stimuli containing features associated with different responses than to stimuli whose features are not inconsistent [3,4]. Gratton et al. [2] reported that this conflict effect may be modulated by *expectancies* about whether the presence or absence of conflict will occur. Conflict adaptation refers to the adjustments that are made based on expectations during performance on a task involving conflict. Can top-down, expectancy-based control operations in the presence of conflict affect attention weights given to different stimulus features (as originally implied by Gratton et al. [2])? Gratton et al. [2] determined that presenting arbitrary cues statistically associated with different conflict conditions (just prior to each target stimulus) is ideal for testing the sensitivity of conflict adaptation (and, hence, cognitive control).

Another question that has been addressed in previous studies, and is relevant to the current study, is whether or not the subjects are “aware” of the changes in expectations occurring during conflict adaptation. This question addresses the issue of whether cognitive control (and cognitive operations in general) is “conscious” or not. Investigators have used two ways of addressing this question. Studies have investigated whether sub-liminal cues (or context changes) can lead to conflict adaptation. The results of these studies have been mixed, although a majority of the reports have shown that sub-liminal stimuli can elicit conflict adaptation. The second approach, which will be used in the current study, is to compare conflict adaptation effects in groups of subjects that, at the start of the study, were explicitly told or not explicitly told about the significance of the cues with respect to whether the upcoming stimulus will involve response conflict or not [5]. Note that with this procedure it is necessary to determine whether, during the study, the subjects became aware of the significance of the cues. This needs to be determined using tests of implicit and explicit knowledge conducted at the end of the study. This second approach, compared with the first approach, more directly tests the issue of whether explicit versus implicit knowledge mediates conflict adaptation. In our previous work we showed that subjects exhibit conflict adaptation without explicit knowledge of the significance of the cues [5]. In the current study we will attempt to replicate this finding, and to determine whether processes of associative learning (competition between cues, such as associative “blocking”) involved in conflict adaptation operate in a similar fashion under implicit learning conditions. Competition between cues has been critical in the development of associative learning and has helped to dissociate theories of learning (e.g., [6–9]). A demonstration of associative competition in the present task will be the first to show competition between cues that can control conflict adaptation strategies.

Therefore, the present study sought to explore whether stimuli would compete in a learning task involving a choice reaction time task and response conflict. The experiments used a procedure in which several different trial types were interspersed during a single phase of training (akin to the “relative validity” procedure used by Wagner [10]) to examine cue competition. There were two goals of the present study. First, the study tested the hypothesis that cue competition occurs for a task that has been shown to involve implicit processes. That is, performance can occur based on the information provided by cues and the participant presumably is not aware of this information. Second, assuming competition occurs, it is not clear if competition will occur for cues that predict compatible trials and cues that predict incompatible trials, or both kinds of cues. It is hypothesized that competition may occur when two cues that predict a compatible trial are presented, given that competition has been shown in many dozens of published works with respect to cues that predict an outcome’s occurrence (here, compatible trials). Demonstrations of competition in predicting the absence of an outcome are rare at best. Therefore it is expected that competition will be less likely to be generated after the presentation of cues predicting incompatible trials. It is not clear that the two types of information (when compatible versus incompatible trials are predicted) are processed similarly; it has been claimed that different processes underlie learning that involves negative correlations and that which involves positive correlations (e.g., [11, p. 1650; 12, p. 1344]).

## Experiment 1

### Materials and Methods

#### Participants

Twenty four students (11 women and 13 men) at the University of Missouri received course credit for their participation in this experiment. The age of the subjects ranged from 21 to 27 years old and they had normal or corrected-to-normal vision and hearing.

#### Stimuli and Procedure

Two stimuli were presented on a computer screen on each trial of an Eriksen flanker task [4]. Participants sat approximately 1 m from the computer monitor with their eyes level and fixated on the screen’s center. To minimize visual search, a fixation cross was placed at the center of the computer screen and remained visible at all times during the experiment. An experimental trial consisted of the following events: first, the cue stimulus (A, B, C, D, or the combination AC or BD) was presented on the screen for 100 ms. Similar to the procedure used by Gratton et al. [2], the second target stimulus consisted of one of four 5-letter arrays (HHHHH, SSHSS, SSSSS, or HHSHH) and the middle letter of the array was designated as the target, and the other letters (noise letters) were used as distractors. The subjects were instructed to respond, using a computer keyboard, to one of the possible target letters (H or S, the letter in the middle of the 5-character array) by pressing a certain key on the keyboard with one hand. They were told to respond to the other letter in the middle of the array by pressing a different key on the keyboard with the other hand. The letter hand-association was counterbalanced across the subjects in each group. The visual angle subtended by each letter of the array was approximately 0.5 degrees. The angle subtended by the whole array was approximately 2.5 degrees. When the pre-trial cue stimulus was a single letter, this letter was presented on the left or right side of the fixation cross. When the cue stimulus was a combination of two letters (AC or BD), both letters were presented simultaneously on the screen: one to the left and the other to the right of the fixation cross. The letter position (single cue and compound cue trials) was counterbalanced across subjects. Next, the cue stimulus disappeared leaving the fixation cross on the screen for 1500 ms. Then, the array (HHHHH, HHSHH, SSSSS, or SSHSS) was presented for 100 ms. The target letter (H or S) was located above the fixation cross.

Each subject was tested individually and completed two sessions. The first session was considered practice, and involved 5 blocks of 30 trials. In the second (experimental) session the subjects completed 64 blocks of 30 trials. The subjects were verbally instructed to respond as quickly as possible without making more than 10% to 20% errors in a block of trials, and they were asked (between trial blocks) to respond faster if their error rate was less than 10%.

Two (within-subjects) conditions were created: one in which the target letter had the same response as the noise letters (HHHHH or SSSSS; compatible-noise condition), and another in which the noise letters were associated with the alternative response from that indicated by the target letter (HHSHH or SSHSS; incompatible-noise condition). In this situation, the incompatible-noise condition typically leads to slower responses and larger error rates than the compatible-noise condition [4]. The difference in reaction times between the two conditions is usually attributed to involuntary processing of the noise letters and to response competition on incompatible trials. If the incorrect response is activated by the noise letters being processed, then this will compete with the correct response, thus a longer time will be needed to initiate the overt response, and more errors will occur. These effects of the noise are called “noise compatibility effect” or “flanker effect”. Specifically, the noise compatibility effect refers to a difference in performance on compatible and incompatible trials: the occurrence of long latencies (and more errors) on incompatible trials relative to compatible trials. In fact, the effect is calculated as the numerical difference between the typically short latencies (or few errors) on compatible trials (C) and the longer ones (or more errors) on incompatible trials (I, hence I–C).

Gratton et al. [2] and Ghinescu et al. [5] showed that subjects’ expectancies for compatible or incompatible trials can be manipulated by presenting cues (e.g. A, B, AC, BD) that predict the type of upcoming trial. Contingencies were established between particular cues and the probability of occurrence of a compatible or incompatible trial. When subjects are led to expect that a compatible-noise condition will occur, they use a strategy leading to fast responses, but also lead to a high susceptibility to distractor information (which can lead to errors on the relatively rare occasions when the target and noise do not match). When subjects are led to expect that incompatible-noise conditions (i.e., conditions in which target and noise letters are associated with conflicting responses) will occur, they use a strategy leading to slow responses, but also to low susceptibility to distractor conditions. Based on previous research [2], it was expected that subjects would use the information provided by the cues to improve their performance, and would be able to vary their strategies as a function of the cue. The actual letters (A, B, C, D) were counterbalanced with respect to the predictive role that they possessed, but we use the combinations described below as our example throughout this paper. For example, for some participants, Cue A and also Cues AC (as a stimulus compound) predicted a compatible trial. Cue B and also the compound BD predicted an incompatible trial. When a cue (A, AC) predicted a compatible trial, it indicated a 0.8 probability that the trial following it would be “compatible” (HHHHH or SSSSS). The stimulus compounds B and BD both indicated a 0.8 probability that the subsequent trial would be “incompatible” (HHSHH or SSHSS). On probe trials in which the cue was either C or D alone, there was a 0.5 probability that the following trial was a compatible trial and a 0.5 probability that it was an incompatible trial. Note that C was present on AC trials for which there was an 80% chance of a compatible trial (and 20% incompatible), and when it was presented alone on probe trials the probability of a compatible trial was 50% (and 50% incompatible). Since the number of C alone trials was the same as the number of AC trials during training, the overall probability of a compatible trial given the presence of C on the trial was 65%. The probability of an incompatible trial given C was therefore 35%. A similar relationship existed for D on BD trials in which there was an 80% chance of an incompatible trial and D alone probe trials also occurred (with a 50% probability of each outcome). Overall, D had a 65% chance of being followed by an incompatible trial and a 35% chance the trial was compatible. The time allocated for responding on each trial was 1500 ms [2, 5], which is sufficient for participants to evaluate the cue, select a strategy, and prepare to use this strategy. The intertrial interval was 2000 ms.

Gratton et al. [2] showed that subjects could use different strategies to perform this task: a “parallel strategy” (responding on the first overall impression of the stimulus array), a “focused strategy” (focusing on the central letter), and a “guessing strategy”. It was hypothesized that when a parallel strategy is used, the noise-compatibility effect on reaction time will be large and accuracy will be poor (because distractor noise stimuli are used and such information can lead to an incorrect response). It was also hypothesized that when a focused strategy is used, the noise-compatibility effect on reaction time will be low because all times will be slow.

It was expected that training with the A cue alone as a predictor of a compatible trial (with 80% probability) would reduce, through cue competition [9, 10], the degree in which C would be learned about as a stimulus that predicts a compatible trial. Similarly, it was expected that the B-alone training trials would reduce the degree in which cue D would be learned about as a predictor of incompatible trials. It was therefore expected that the A cue would produce a large noise compatibility effect (evidence of a parallel search process) and the B cue would produce a low noise compatibility effect (evidence of use of a focused search). For the B cue, RT and error scores should be large since incompatible trials were frequent. If the C and D cues are not learned about, it was expected that they would induce a parallel search. Parallel search is common for uninformative or novel stimuli [2, 13], and a focused search must be acquired for events that would warrant such a strategy adaptive (e.g., if an incompatible trial is likely).

The cues were randomly ordered within each block of trials, with the constraint that a specified number of each trial type occurred. Each of the predictive cues (A, B, AC, and BD) occurred on 16.7% of the trials, and each of the critical test cues (C and D) also occurred on 16.7% of the trials. The overall probability of compatible or incompatible trials was 0.5 for all trials combined.

In order to explore the possibility that strategy selection can be learned implicity, participants were divided into three equal-size groups and the groups received different instructions. At the beginning of the experiment the subjects in the first group (Explicit Instructions Group) were informed about what the cues predicted (with respect to the probability of the following trial being compatible or incompatible) and they were told to use this information when responding. For example, they were told that Cues A and AC predicted a compatible trial 80% of the time, and Cues B and BD predicted an incompatible trial 80% of the time. The other cues were described in this way as well. In particular, they were asked to respond to the first overall impression of the stimulus array when they expected a compatible trial (parallel strategy), to focus on the central letter when an incompatible trial was expected (focused strategy), and to use any strategy when a compatible and incompatible trial were expected to be equally likely.

The subjects in the second group (Partially-Explicit Instructions Group) were only informed that the cues had *some* predictive value. Specifically, they were told that the cues predicted whether the array that followed would be compatible or incompatible. No information about the probabilistic value of the cues was given. The subjects were told that by being exposed to many trials with the cues and the arrays of letters that followed the cues, they should be able to predict what kind of condition would occur and, consequently, they should be able to use this information when responding. Similar to the first group, they were instructed to respond to their first overall impression of the stimulus array when they expected a compatible trial, to focus on the central letter of the array when an incompatible trial was expected, and to use any strategy when a compatible and incompatible trial were equally expected.

The subjects in the third group (Implicit Instructions Group) were told that a symbol would precede each occurrence of a stimulus array and that their task was to respond to the central letter of the stimulus array. These subjects did not receive any other information. If strategy selection is learned implicitly, then the type of instruction should not influence the size of the strategic adjustments made by the participants; if, instead, strategy selection requires explicit knowledge, the type of instruction may have an effect. All participants gave informed, written consent regarding participation prior to the start of the experiment. This research project was approved by the University of Missouri Campus Institutional Review Board (Approval Number 1071138).

### Results and Discussion

The data collected during the practice session (and the practice session of Exp 2) were not analyzed. Performance on all experimental trials were used in the analyses below. The results found evidence of competition between cues that predicted an incompatible trial.

#### Reaction time

**Fig 1:**
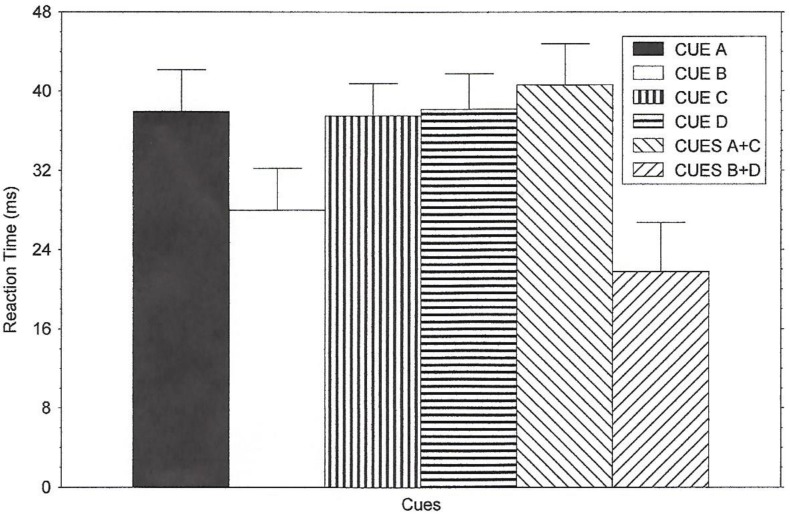
Reaction Time Results of Experiment 1. The figure illustrates the results of Experiment 1 regarding the noise compatibility effect on reaction time (larger scores indicate faster reaction trials on compatible trials than incompatible trials) for all of the cues. The means were obtained by averaging noise compatibility effect scores across the three instruction conditions.

**Fig 2:**
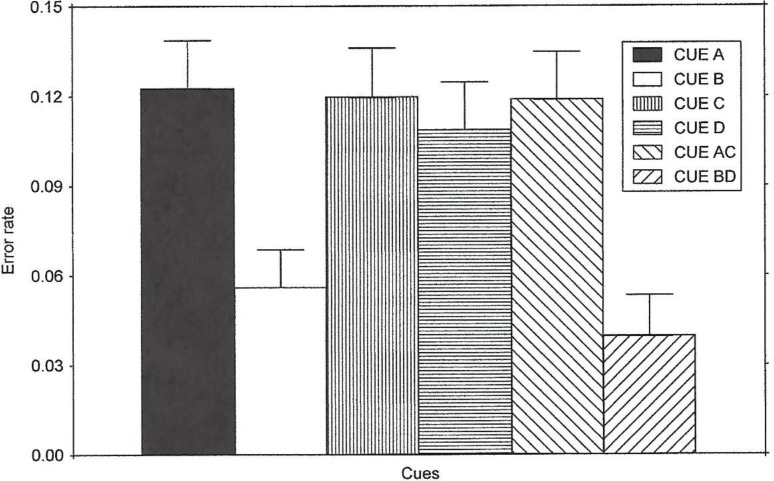
Error Rate Results of Experiment 1. The figure illustrates the results of Experiment 1 regarding the noise compatibility effect on error rate (larger scores indicate greater error rate on incompatible trials than compatible trials) for all of the cues. The means were obtained by averaging the noise compatibility effect scores across all instruction conditions.

Twelve mean reaction times during the experimental session were obtained for each subject: 6 cues (A, AC, B, BD, C, D) x 2 array conditions (compatible and incompatible). All reaction time and error scores are presented as noise compatibility effects [2,5], unless mentioned otherwise, and are shown in Fig 1. The noise-compatibility effect with reaction time was computed as the difference between the reaction times for compatible trials and incompatible trials. This yielded six estimates of the noise compatibility effect on reaction time for each subject (one estimate for each cue type). These group mean scores are shown in Fig 1. The estimates obtained for each subject and condition were entered into a mixed-design ANOVA, with instruction as a between-subjects variable, and cue as a repeated-measures factor, and the noise compatibility effect as the response measure. The main effect of instructions on reaction time was not significant, *F* < 1, η^2^ = 0.0178. The Instruction x Cue interaction was also not significant, *F*(10, 105) = 1.66, *p* >.05, η^2^ = 0.1083. Means and SEMs for reaction times and errors for the three instruction conditions are shown in the top half of S1 Table. Figs 1 and 2.

The reaction time results are depicted in Fig 1 for which the data were collapsed across instruction conditions. However, cue condition affected reaction time performance as revealed by a main effect of this factor, *F*(5, 105) = 5.94, *p* < .0001. Post-hoc contrasts (pairwise comparisons) found that, for the instruction groups collapsed, when the cue predicted a compatible noise trial (Cue A), a larger noise-compatibility effect was observed than when the cue predicted an incompatible noise trial (Cue B), *F*(1, 21) = 9.28, *p* < .05. In the presence of Cue B, subjects were relatively slow on both trial types (compatible and incompatible) since an incompatible trial was presumably expected. In the presence of Cue A, however, participants responded quickly on compatible trials and slow on incompatible trials. Also, a larger noise-compatibility effect was observed for Cue AC (which predicted compatible trials) than Cue BD (which predicted incompatible trials), *F*(1, 21) = 12.90, *p* < .05.

Performance for Cue C did not differ significantly from that of Cue A or Cue AC; both post-hoc contrasts showed no significant difference between the means, *F*s < 1, η^2^ = 0.0007 and 0.0453, respectively. Therefore, Cue C could be viewed as having gained control over responding (i.e., predicting compatible trials) in that evidence of a parallel search process was obtained (a large flanker effect was observed). However, as mentioned, cues that have not been learned about invoke a parallel processing mode such that a noise compatibility effect occurs [2, 5, 14–15]. Learning about Cue C may have actually been blocked by Cue A, and this lack of learning produced quick responses on compatible trials due to a default parallel processing strategy. Alternatively, C may have been learned as a predictor of compatible trials (blocking did not occur). Cue C differed from the cues that predicted an incompatible trial; Cue C differed from Cue B, *F*(1, 21) = 7.37, *p* < .05, and Cue C differed from Cue BD, *F*(1, 21) = 12.24, *p* < .05. Figs 1 and 2. S1 and S2 Tables.

In sum, performance to Cue C was comparable to Cue A. Reaction time performance for Cue D did not produce evidence of this cue predicting incompatible trials (as *was* true for Cue B and Cue BD). Cue D did not differ from Cue C and Cue AC, *F*s < 1, indicating that Cue D produced a parallel search strategy. Cue D differed significantly from the predict-incompatible noise condition cues of B and BD [Cue B, *F*(1,21) = 6.81, *p* < .05, and Cue BD, *F*(1,21) = 11.99, *p* < .05]. S2 Table shows reaction times on compatible and incompatible trials (the scores used in calculating noise compatibility effect values) for each condition.

#### Accuracy

The error data were extremely consistent with the reaction time data. The average error rate for each group and type of cue was calculated in a similar manner to that described for reaction time. The repeated-measures ANOVA revealed that the instructions given to the subjects did not have a significant effect, *F* < 1, η^2^ = 0.0522. There was no significant Instruction x Cues interaction, *F* < 1, η^2^ = 0.0256. The cues affected the accuracy of responses as revealed by a main effect of Cues, *F*(5, 105) = 14.10, *p*< 0.0001. The results of the error rate data were averaged across instruction condition and are presented in Fig 2.

The noise compatibility effect (i.e., greater errors on incompatible than compatible trials) was larger for the cues that predicted a compatible trial than those that predicted an incompatible trial. The contrast between Cue A and Cue B resulted in a group mean difference, *F*(1, 21) = 16.00, *p* < 0.0001, as did the contrast between Cue AC and Cue BD, *F*(1,21) = 20.93, *p* < .001.

The greater effect of noise in the predict-compatible condition was due to an increase in the frequency of errors on incompatible trials when a compatible trial was predicted (those 20% of the trials). Similar to the reaction time measure, the error rates suggested that a parallel search strategy was used in the presence of the C and D cues, and error rates on these trials were similar to those of Cues A and AC. Comparisons between Cues C and A, Cues C and AC, and Cues D and AC all resulted in *F*s < 1, three η^2^ = values were 0.0337, 0.0002 and 0.04101, respectively. When Cue C was compared to Cue B, the effect of noise on error rate was significant such that Cue C resulted in more errors, *F*(1, 21) = 23.02, *p* < .0001. Cue C also produced more errors than Cue BD, *F*(1,21) = 29.55, *p* < .0001. Cue C resulted in performance like that which is based on parallel search; and this may have been because the cue predicted a compatible trial (no blocking occurred), or it may have been because it was not learned about (since novel cues are known to elicit parallel search, and therefore many errors occur).

Similar to the effects observed for the reaction time data, Cue D differed from both Cue B and Cue BD, *F*(1,21) = 17.84, *p* < .0001, and *F*(1,21) = 22.49, *p* < .001, respectively. Cues C and D did not differ, *F*(1, 21) = 1.73, *p* > .05, nor did Cues D and A, *F*(1, 21) = 1.67, *p* > .05. Similar to the results obtained with reaction time, the error rate data for the C and D cues resembled those of a predict-compatible condition. This is again consistent with the idea that the subjects used a parallel strategy for the redundant cue (D) condition when it was presented alone on probe trials. Cue D did not appear to be learned about as a predictor of incompatible trials. Learning about it was blocked due to its redundant status. The results reveal competition among cues that predict incompatible trials. The expectation that an incompatible trial will occur results in conflict adaptation–an adjustment in processing strategy in preparation for the predicted trial type. Although this adaptive process is an implicit one [5], cues appear to compete in their ability elicit the conflict adaptation strategy (at least with respect to the prediction of incompatible trials). The possibility of competition during predict compatible trials is less clear. Again, because subjects produce parallel search to unlearned cues, C may have been kept from acquiring signaling value due to competition. S2 Table shows the mean percentage of trials in which the correct response occurred on compatible and incompatible trials (the scores used in calculating noise compatibility effect values) for each condition.

## Experiment 2

Experiment 1 was a small scale, initial study which found that the instruction conditions did not impact behavioral control by the cues. Experiment 2 further examined if the selection of strategies occurs at an implicit or explicit level of awareness. Experiment 2 was a replication of Experiment 1 with two added measures of awareness.

### Materials and Methods

#### Participants

Thirty-nine students (22 men and 17 women) at the University of Missouri participated for course credit. The subjects ranged in age from 19 to 25 years and they had normal or corrected-to-normal vision and hearing.

#### Stimuli and Procedure

Following the practice trials, all subjects received an initial phase of training. The subjects were randomly assigned into three equal-sized instruction groups, and the subjects received exactly the same instructions as in Experiment 1. After completing the experimental procedure that was identical to that of Experiment 1, the subjects were asked to fill out two questionnaires to assess the participants’ explicit knowledge of the information provided by each cue. The first questionnaire was a forced-choice task. Each subject received a list of 72 questions. A cue (i.e., A, B, C, D, AC, and BD) was presented along with two arrays of letters, such as those that could have followed the cue during the earlier training. The subjects were asked to choose which of two sample, 5-character stimulus arrays were more likely to follow the cue (or combination of cues). Each cue could be followed by a total of six combinations of arrays (HHHHH with either SSSSS, HHSHH, or, SSHSS; SSSSS with either SSHSS or HHSHH, and HHSHH with SSHSS). For each cue, these six combinations were used a second time with the choice order inverted. Thus, a total of 72 possible combinations were used (6 cues x 12 array combinations).

The second questionnaire asked the subjects to indicate on a scale from 0 to 100 (graded in 10 unit intervals) what percent of the time a given cue or combination of cues was followed by a certain target stimulus. A total of 24 questions was used with each cue followed by one of the four possible arrays of letters. Half of the subjects completed the forced choice task first, and then the probability estimation, whereas the other half completed the two tasks in the inverse order. All unspecified details were the same as in Experiment 1.

### Results and Discussion

The results found, consistent with those of Experiment 1, that competition occurs between cues that predict an incompatible trial.

#### Reaction time

**Fig 3:**
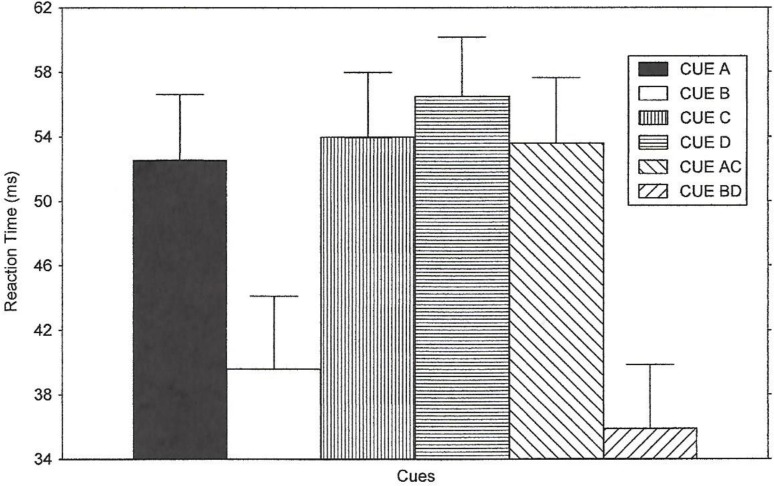
Reaction Time Results of Experiment 2. The figure illustrates the results of Experiment 2 regarding the noise compatibility effect on reaction time for all of the cues. The means were obtained by averaging the noise compatibility effect scores across all instruction conditions.

The main effect of instructions was not significant, *F* < 1, η^2^ = 0.0163. The Instruction x Cue interaction was also not significant, *F*(10,180) = 1.50, *p* >.05, η^2^ = 0.0595. The noise compatibility results are presented in Fig 3 for which reaction times were averaged across the instruction conditions. Means and SEMs of the noise compatibility effect for the error rates for the three instruction conditions are shown in S3 Table.

Cue condition affected reaction time performance, *F*(5,180) = 10.43, *p* < .0001. Post-hoc contrasts found that for all three instruction groups collapsed, Cue A produced a larger noise-compatibility effect than Cue B, *F*(1,36) = 10.96, *p* < .001. Also, a larger noise-compatibility effect was observed when comparing Cue AC than Cue BD, *F*(1,36) = 23.23, p < .001. This larger effect of noise on reaction time in the predict-compatible condition (Cue A, Cue AC) was due to faster responses on the compatible trials.

Similar to the results of Experiment 1, the group mean noise compatibility effect for reaction time for Cue C did not differ from that of Cue A, *F* < 1, η^2^ = 0.0050, or Cue AC, *F* < 1, η^2^ = 0.0003. Hence, no apparent reduction in control by C was produced; Cue C did differ from Cue B, *F*(1,36) = 9.26, *p* < .0044, and Cue C also differed from BD, *F*(1,36) = 18.0, *p* < .0001. Like the results of Experiment 1, Cue C apparently produced a parallel search process. This could indicate that Cue C was learned about as a predictor of compatible trials, although parallel search is common for novel or unlearned stimuli [2].

**Fig 4:**
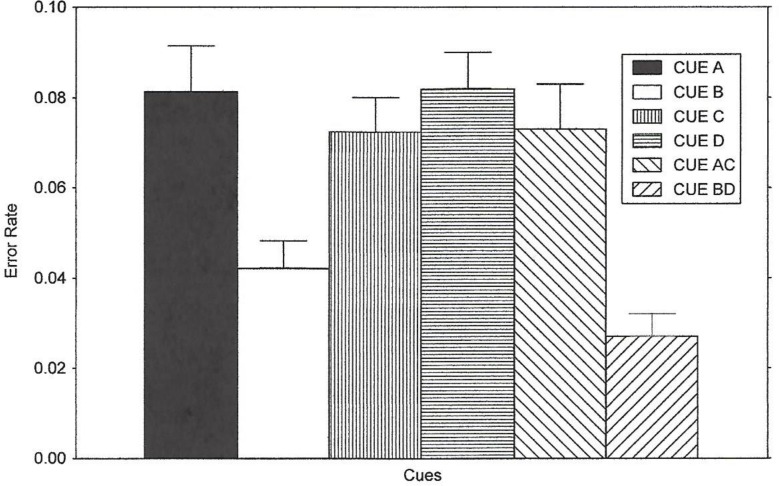
Error Rate Results of Experiment 2. The figure illustrates the results of Experiment 2 regarding the noise compatibility effect on error rate for all of the cues. The means were obtained by averaging the noise compatibility effect scores across all instruction conditions.

Cue D did not come to predict incompatible trials as determined by the magnitude of the noise compatibility effect for this condition. Cue D did not differ from Cue A, *F*(1,36) = 1.70, *p* >.05, nor Cue AC, *F* < 1. Cue D did differ from Cue B, *F*(1,36) = 16.91, *p* < .0002, and Cue D differed from Cue BD, *F*(1,36) = 26.15, *p* < .0001. Cues C and D did not differ, *F* < 1. The noise compatibility effect for the B and BD cues were smaller than those in Experiment 1. Similar to the results of Experiment 1, Cues C and D closely resembled those of the predict-compatible conditions (A and AC). Cues C and D apparently elicited a parallel strategy. That is, following the presentation of these cues, the subjects used the noise information to make fast responses on compatible trials, even if such reaction times were slow on incompatible trials. Cues B and BD produced a focused strategy, while performance to Cue D was consistent with a parallel strategy. Reaction times on compatible and incompatible trials (the scores used in calculating the noise compatibility effect values) are presented for each condition in S4 Table. Figs 3 and 4. S3 and S4 Tables.

#### Accuracy

Fig 4 shows error rates averaged across the instruction conditions. The repeated-measures ANOVA revealed that the instructions given to the subjects did not have a significant effect on accuracy, *F*(2,36) = 3.01, *p*>.05, η^2^ = 0.1434. There was a significant Instruction x Cue interaction, *F*(10,180) = 2.98, *p* < .05, η^2^ = 0.0957. This was the first effect obtained in this study in which the factor of instructions was involved. This occurred because the noise compatibility effect for the predict compatible conditions (Cues A, C and AC) were somewhat larger for the Explicit condition than the Partially-Implicit or Implicit conditions. Awareness of the probabilities likely produced a stronger tendency for parallel search in the Explicit condition.

The cues affected the accuracy of responses, *F*(5,180) = 14.40, *p*< 0.001. A contrast analysis of the effect of noise on error rate showed that the noise compatibility effect was larger for Cue A than Cue B, *F*(1, 36) = 13.70, *p* < 0.001, and for Cue AC relative to Cue BD, *F*(1,36) = 27.02, *p* < .001. As with the earlier results described, the greater effect of noise in the predict-compatible conditions was due to an increase in the frequency of errors on incompatible trials (the 20% of the trials in which incompatible noise occurred).

As occurred for the reaction time measure and similar to the results above, the noise compatibility effect for Cue C was similar to that for Cues A and AC, *F*(1, 36) = 1.11, p > .05 and *F* < 1, respectively. The η^2^ values for these comparisons were 0.0298 and 0.00017, respectively. The group mean error rate for Cue C differed from that of both Cue B and Cue BD, *F*(1, 36) = 13.25, *p* < .001, and F(1,36) = 33.02, p < .0001, respectively. Cue C produced a parallel search strategy.

Cue D did not differ from Cue AC, *F*(1,36) = 1.21, *p* >.05, or Cue A, *F* < 1. Error rate performance for Cue D relative to Cue B and Cue BD was significantly different, *F*(1,36) = 20.97, *p* < .0001, and *F*(1,36) = 40.08, *p* < .0001, respectively. Cue C and D did not differ, *F*(1, 36) = 2.49, *p* > .05. Cue D failed to predict incompatible trials as indexed by the noise compatibility effect for this condition. Similar to the results obtained with RT in this experiment and the results of Experiment 1, the error rate data for both Cues C and D resembled those of predict-compatible conditions. This is again consistent with the idea that the subjects used a parallel strategy for these cues on the critical test probe trials, and Cues B and BD elicited focused search. Cue D did not appear to be learned about during training. Mean percentage of trials in which the correct response occurred on compatible and incompatible trials (the scores used in calculating noise compatibility effect values) are presented for each condition in S4 Table.

#### Measures of awareness data

**Fig 5:**
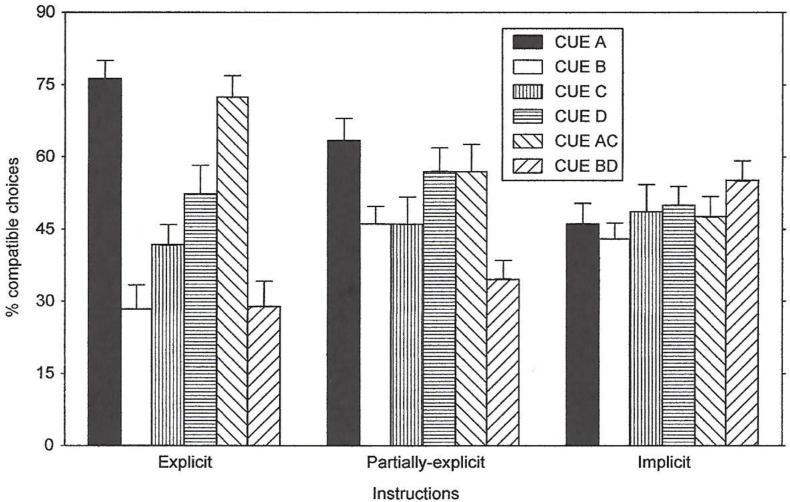
Questionnaire Data (Percent Compatible Choices) of Experiment 2. Percent compatible choices in a forced-choice task given by the explicit, partial-explicit, and implicit instructed subjects for all of the cues.

**Fig 6:**
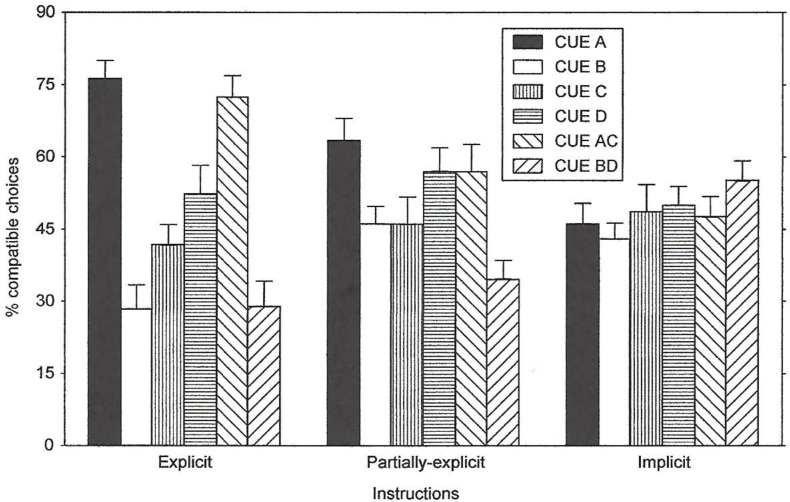
Questionnaire Data (Probability Estimation) of Experiment 2. Percent compatible choices in a probability-estimation task given by the explicit, partial-explicit, and implicit instructed subjects for all of the cues.

Two measures were used to assess the subjects’ expectations about the predictive value of the cues: a forced choice questionnaire and a probability estimation questionnaire. The results for the forced choice task are shown in Fig 5. The subjects in the Explicit Group remembered and understood the instructions given before they began the experiment. The subjects in the Partially-Explicit and Implicit groups appeared to lack awareness of the predictive value of the cues. The results of a repeated-measures ANOVA obtained a main effect of Cues, *F*(2,36) = 12.54, *p* < .0001. As expected, the Instructions x Cues interaction was also significant, *F*(10,180) = 5.92, *p* < .0001. Given the instructional manipulation, some conditions were aware of the contingency, while others were not. Figs 5 and 6.

The results for the probability estimation task are depicted in Fig 6. Similar to the results of the forced choice task, the subjects in the Explicit group showed explicit knowledge of the predictive value of the cues on A and AC trials. The other two groups showed little explicit knowledge about the cues’ predictive value. For these two groups the values approached the 50% value, with the exceptions that the Cue B and Cue BD responses appeared below chance.

## Conclusions

The purpose of this paper was to determine whether strategy selection is regulated by previous experience, in a manner consistent with what is known about associative learning. Associative learning theories claim that competition should occur between cues. Specifically, we were interested in determining whether the adaptive learning of strategy selection shows evidence of cue competition. Since this adaptive strategy selection has been shown to involve implicit processes, we asked two other questions: Will cues compete during this type of learning and will competition occur for cues that predict the occurrence of compatible trials and for those that predict incompatible trials? Will strategy selection based on an added, redundant cue (e.g., cue C or D in the present case) be influenced by trials in which cue A alone predicts a compatible trial and cue B predicts an incompatible trial? We would consider such a finding as evidence that metacognitive processes such as strategy selection are regulated by similar mechanisms to other types of more elementary cognitive processes. Indeed, there are reasons to claim that control during a conflict adaptation task can stem from an associative process [16].

To address this issue we employed a variant of Eriksen’s flanker paradigm [4] adopted in previous studies (e.g., [2, 5]). Earlier work [13] showed that subjects, when confronted with noisy stimuli such as those used in the flanker task, process information in one of two ways. Parallel processing is unselective and therefore largely influenced by the noise conditions (i.e., compatible or incompatible noise). Focused processes are more selective and little affected by the noise conditions. The type of processing employed to activate the subject’s response is, however, under strategic control [2]. When subjects expect a compatible trial based on evidence accumulated during parallel processing, their responses on compatible noise trials will be fast; however this information is not selective and incompatible noise may generate large amounts of response conflict. In this case, the difference between the reaction time and accuracy on compatible and incompatible trials (the “noise effect”) would be large. The “focused” strategy leads to responses whose RT and accuracy are less affected by the noise level.

According to this logic, strategy selection is consistent with a subject’s analysis of the costs and benefits associated with each choice: Subjects choose a strategy that helps them improve performance by making their responses faster (parallel strategy) or by avoiding errors (focused strategy). If experimental conditions are set up so that the probability of compatible or incompatible noise on a particular trial can be predicted, the subjects will adjust their strategy, and the size of the flanker effect will vary. Although some debate still exists regarding the occurence of conflict adaptation in conditions in which the objective probability of compatible and incompatible noise does not vary across trials (e.g., [17]), we use here a task in which an arbitrary cue signals this probability on a single trial basis; our previous studies show robust evidence for conflict adaptation under these conditions (e.g., [2, 5]).

Since we were interested in how subjects learn to adapt to different experimental conditions, we used several types of cues. Two of the cues (A and B) predicted what type of trial (compatible or incompatible) was likely to follow, and therefore provided information about the best strategy to use across the duration of the experiment. For example, when cue A predicted a compatible trial, subjects could use a “parallel” strategy, whereas when cue B predicted that an incompatible trial was likely to follow subjects could use a “focused” strategy. The variations in the flanker effects on RT and accuracy across these two prediction conditions indicated that subjects actually used the information provided by the cue and adopted the strategy that was more likely to help them improve their performance.

The results obtained with Cues A and B are consistent with earlier work using cues to demonstrate conflict adaptation [2, 5]. Note that we can construe the two stimuli (cue and the target array) in the cued flanker task as an associative learning task in which a conditioned stimulus or CS (the cue) occurs with a target array as the unconditioned stimulus (US) outcome. The conditioned response, in this way of interpreting this paradigm, is represented by the strategic choice by the subject (i.e., employing a “parallel” or a “focused” strategy in response to the US), and evidence of its occurrence is the modulation of the flanker effect (i.e., the conflict adaptation effect). In this sense, the results obtained with Cues A (“predict compatible”) and B (“predict incompatible”), a modulation of the conflict effects expressed in RT and accuracy, indicate that subjects learned the association between the CS and the US (as it was also demonstrated in [2] and [5]).

In the current study, in both Experiment 1 and 2 we added other types of cue conditions to demonstrate that cue competition may also occur in this paradigm. The results were very clear in supporting our hypothesis that cue competition could occur between cues that instruct what kind of strategy to use. However, definitive evidence of cue competition could only be obtained in some conditions but not in others. In this sense, our hypothesis was not supported: it was expected that cue competition would occur between “predict compatible” cues and “predict incompatible cues”. In fact, since competition in associative learning studies is extremely prevalent among published studies in which cues predict an outcome, while competition in associative learning is rare for cues that predict the absence of an outcome, the opposite outcome to the present findings was expected. We used a single phase of training and a within-subject design in which six types of cues were presented: four types of simple cues (A = “predict compatible”, B = “predict incompatible”, C and D = “no prediction”) along with two compound cues (AC = “predict compatible”, and BD = “predict incompatible”). We found evidence of competition between B and D but not A and C. The A cue became a fairly valid predictor that compatible trials would follow. The B cue became a valid predictor that incompatible trials would follow. The redundant D cue accompanied this B cue on some trials. In contrast to the behavior produced by Cue B (behavior consistent with a focused search strategy), the D cue failed to generate the capability to predict incompatible trials and a resulting focused search. Cue D produced a statistically *different* degree of control over behavior than the B cue or the BD cue (see Figs 3 and 4). Because it can be assumed that the D stimulus would have acquired predictive value (i.e., predicting incompatible trials) had it been trained in isolation (as B had), then we must conclude that the B cue influenced the manner in which D was learned about. The differences in reaction times and error results were virtually identical in the two experiments.

The first experiment revealed an instance of cue competition. Notice that in this experiment, different instructions were given to three groups of subjects about the predictive roles of the cues. One group of subjects was given explicit instructions about the significance of cues A and B (“explicit instruction” group). A second group was only told that these cues were related to the probability of the compatible and incompatible noise, but not their specific value (“partially explicit instruction” group). A third group was told nothing about the relationships between the cues and the target array (“implicit instruction” group). In previous work, we showed that the behavior of the subjects was similar across these instruction conditions, suggesting that explicit knowledge about the significance of the cues is not required for showing conflict adaptation [5]. In that study we used a specific procedure to investigate the level of subjects’ explicit knowledge of the cue meaning at the end of the experiment, and exclude the possiblity that the implicit group might develop explicit knowledge across the duration of the experiment.

To document that any learning occurring in the current experiment was implicit, in Experiment 2 we replicated the learning procedure used in Experiment 1 employing the same procedures used in Ghinescu et al. [5] to document the type of explicit knowledge the subjects had about the predictive value of the cues at the end of the experiment. The results of the experiments showed that subjects in the Implicit and Partially-Explicit groups were able to select strategies during the AC and BD compound cues as well as the A, B and C cues, and use them when responding, even in the absence of explicit knowledge about the meaning of the cues at the end of the experiment. The Explicit group, of course, used appropriate strategies after receiving explicit instructions.

In both Exp 1 and 2, whereas cue D was blocked by cue B, Cue C was not blocked by Cue A. This asymmetry in cue blocking may reflect an asymmetry between the two strategies: One of the two processing strategies may be used as a “default” strategy, to be used for most stimulus conditions, and the other as a “special” strategy, to be used only for particular situations. Indeed, an asymmetry between the “predict compatible” and the “predict incompatible” condition could be predicted by theories such as the “conflict monitoring hypothesis” [18]. This theory envisions a revision of processing weights favoring a more selective (“focusing”) processing strategy for conditions in which incompatible noise conditions are expected–whereas all other conditions might merely lead to a relaxed state such that a default, parallel mode of processing occurs. Similarly, subjects may use a parallel strategy for novel cues or cues that have yet to acquire signaling value [2, 5]. Parallel search is common for uninformative or novel stimuli, while a focused search must be acquired for events that would warrant such a strategy (e.g., if an incompatible trial is likely).

The present findings also have implications for the type of processing that occurs during a cue competition procedure. Although we describe the processing underlying these effects as due to implicit strategic decisions, it is valuable to consider these findings in light of associative learning theories. Views of associative learning, such as the Rescorla-Wagner model [9], claim that competition for associative strength occurs. These associative learning theories cannot explain why competition should occur for situations in which cues predict incompatible trials, but a lack of competition on “predict compatible” trials. They also cannot address the finding of competition between cues that subjects have learned are uninformative (“learned irrelevance”, see [19]). The present demonstration of competition using an implicit, automatic process has been obtained in an earlier study [20].

The conflict monitoring system likely responds differently when a compatible trial is expected as a result of the pretrial cue, and when an incompatible trial is expected based on the pretrial cue. The conflict monitoring process involves an evaluative processing phase when response conflict is detected; and this is followed by a regulatory processing phase in which control processes attempt to initiate the appropriate response in trying to adapt to conflict. The evaluative phase has been claimed to involve amygdalar structures as well as orbitofrontal and insular cortices (see [21] for a review). Regulatory processes are said to involve the anterior cingulate cortex (ACC) [18,22]. When an incompatible trial is expected, the regulatory process and ACC likely have already been activated when the array is presented. When a compatible trial is expected, the array first initiates activation of the evaluative process. It is not known why blocking should occur for cues that predict incompatible but not compatible trials using the present procedure, but the fact that processing is not identical in these situations makes this difference less surprising. Different processes appear to exist for situations that involve response conflict and those that do not. For instance, the N200 component of the evoked potential appears to be sensitive to the experience of response conflict [23], while other processes may be involved in consonant events. As mentioned, it has been claimed that different processes may underlie learning that involves negative correlations and that which involves positive correlations (e.g., [11–12]. This makes the finding that dissociation is possible for the processes underlying the prediction of compatible and incompatible trials less surprising.

It should be noted, however, that there is another explanation as to why Cues C and D were not learned about in Experiments 1 and 2 (assuming Cue C was blocked and a parallel search occurred for that reason). It is possible that a unique cue [24–27] was acquired on AC and BD trials. That is, the presentation of A and C together produced a stimulus that was very different from the features of A alone and features of C alone. Therefore, the participant learned about a configural stimulus during training. C and D were not perceived as relevant to training when they were presented alone on probe trials. Although the probe trials (C alone and D alone) probably reduced the likelihood of such configural stimuli (i.e., the presentation of individual elements alone typically disrupt the formation of unique, configural cues), such an explanation cannot be ruled out.

Gratton et al. [2] inferred that top-down control operations during conflict adaption can affect attention weights given to different stimulus features. The present results are consistent with the view that cues (e.g., A) produce control operations that impact the strategy that individuals will use. Some have pointed out that apparent control could be an artifact of the occurrence of stimulus or response repetition [28]. There is disagreement regarding whether conflict adaptation can be obtained—even when trials in which stimulus or response repetition (a potentially confounding variable) are eliminated from the analysis, while other studies have shown no conflict adaptation when this precaution was taken. The current cueing procedure eliminated the confound of stimulus and response repetitions, since the probability of a trial–with respect to it being preceded by a similar or different stimulus or a similar or different response requirement—is always the same for all cue conditions in the present procedure.

It should be noted, however, that a form of priming might be considered as underlying the current effects: It is possible that the cues elicit some form of representation of possible target stimuli containing compatible or incompatible noise–and that the activation of these representations may facilitate their further processing. This could lead to faster RT for compatible arrays following a predict compatible cue and of an incompatible array following a predict incompatible cue. Of course, the data of the current study (as well as those of Ghinescu et al. [5]) suggest that this phenomenon could be occurring without explicit knowledge by the subjects.

The differentiation between this “priming” view and the “strategic” view is related to the specificity of the representations that are “pre-activated,” or facilitated by the cues: According to the priming view, only a very specific set of representation, corresponding to the particular stimuli that are expected to occur, would be facilitated. Whereas the strategic view would assume that all representations of a particular class (i.e., those obtained early during processing or those obtained late in processing) are facilitated together. Interestingly, the asymmetry we observed between the learning processes for predict-compatible and predict-incompatible conditions appears more consistent with the strategic than with the priming hypothesis. In fact, the priming hypothesis would need to explain why the link between cues and the representations for incompatible arrays are learned (and therefore facilitated) in a manner very different from the link between cues and the representations for compatible arrays. The strategic view, instead, can handle this differentiation much more easily, by merely postulating the existence of some hierarchy between different strategies, a view consistent with the conflict adaptation theory [18].

Notebaert and colleagues [16–17] noted that many instances of behavioral control in situations like the present one can be due to habits that arise from stimulus-response repetitions. This effect, although potentially responsible for many results that appear to be due to strategic processing, does not adequately explain the present findings because a zero correlation existed for the specific hand used for responding on the previous trial and that required for the current trial. This was true for trials in which a compatible trial was predicted and trials for which an incompatible trial was predicted.

In summary, the results of the current study indicate that rules of associative learning, such as those leading to cue competition, exert an influence during conflict adaptation in a cueing paradigm. This study provides evidence that metacognitive processes such as strategy selection are regulated by similar mechanisms to other types of more elementary cognitive processes. The present study is the first to examine competitive processes during strategy selection. The results, however, suggest the occurrence of an asymmetry in how these learning rules influence conflict adaptation, with the occurrence of blocking of “predict incompatible” conditions, but not “predict compatible conditions.” This asymmetry is more readily accounted for by accounts that view conflict adaptation as a result of a strategic phenomenon than for those invoking a priming explanation.

## Supporting Information

S1 Table Reaction Times and Error Rates (Noise Compatibility Effects) from Experiment 1 for each Instruction Condition. Percent compatible choices in a probability-**estimation task given by the explicit, partial-explicit, and implicit instructed subjects for all the cues.**
(DOCX)

S2 Table Reaction Times and Error Rates from Experiment 1 for each Instruction Condition. Means (+ SEMs) from Experiment 1
(DOCX)

S3 Table Reaction Times and Error Rates (Noise Compatibility Effects) from Experiment 2 for each Instruction Condition. Percent compatible choices in a probability-**estimation task given by the explicit, partial-explicit, and implicit instructed subjects for all the cues.**
(DOCX)

S4 Table Reaction Times and Error Rates from Experiment 2 for each Instruction Condition. Means (+ SEMs) from Experiment 1
(DOCX)

S1 File Ethics Statement. Ethics Statement.
(DOCX)
