# Supplementary material for: Conflict Adaptation and Cue Competition during Learning in an Eriksen Flanker Task
Source: PLoS One. 2016 Dec 12;11(12):e0167119. doi: 10.1371/journal.pone.0167119 (PMC5152815; doi:10.1371/journal.pone.0167119)
Supplement: S1 Table — Percent compatible choices in a probability-estimation task given by the explicit, partial-explicit, and implicit instructed subjects for all the cues. (DOCX) [file pone.0167119.s001.docx]

Table 1: Means (+ SEMs) from Experiment 1

Reaction Time (Noise Compatibility Effect in Milliseconds)

GROUP CUE

A B C D AC BD

Explicit 39.0+5.06 31.25+6.26 37.13+5.05 37.38+6.73 40.75+6.38 10.00+11.80

Partial Exp 35.13+6.10 24.88+5.63 36.63+6.47 40.38+5.66 29.13+5.26 30.38+6.78

Implicit 39.75+10.27 27.88+9.98 38.75+6.14 36.75+7.04 52.00+8.10 24.88+5.15

________________________________________________________________________________

Errors (Noise Compatibility Effect: Errors on Incompatible Trials – Errors on Compatible Trials)

GROUP CUE

A B C D AC BD

Explicit 0.11+0.03 0.05+0.02 0.11+0.02 0.09+0.02 0.13+0.03 0.02+0.03

Partial Exp 0.11+0.03 0.04+0.02 0.11+0.03 0.10+0.03 0.11+0.02 0.03+0.01

Implicit 0.14+0.02 0.09+0.02 0.14+0.03 0.13+0.03 0.12+0.04 0.07+0.03
