# Supplementary material for: Conflict Adaptation and Cue Competition during Learning in an Eriksen Flanker Task
Source: PLoS One. 2016 Dec 12;11(12):e0167119. doi: 10.1371/journal.pone.0167119 (PMC5152815; doi:10.1371/journal.pone.0167119)
Supplement: S2 Table — Means (+ SEMs) from Experiment 1 (DOCX) [file pone.0167119.s002.docx]

Table 2: Means (+ SEMs) from Experiment 1

Reaction Time on Predict Compatible Trials

GROUP CUE

A B C D AC BD

Explicit 394.0+16.3 384.4+15.2 395.3+16.1 395.4+16.0 392.9.8+15.4 396.5+16.9

Partial Exp 415.5+16.6 423.0+19.0 422.0+16.7 416.3+16.6 409.5+16.6 416.9+15.8

Implicit 409.9+28.8 405.8+30.1 411.1+26.7 409.0+26.9 405.9+24.3 410.3+25.7

________________________________________________________________________________

Reaction Time on Predict Incompatible Trials

GROUP CUE

A B C D AC BD

Explicit 433.0+15.6 415.6+19.0 432.4+16.0 432.8+17.5 433.6+14.6 406.5+21.9

Partial Exp 450.6+17.6 447.9+20.9 458.6+19.5 456.6+17.6 438.6+16.8 447.3+18.4

Implicit 449.6+25.9 433.6+26.9 449.9+27.6 445.8+23.9 457.9+28.7 435.1+26.2

Percent Correct on Predict Compatible Trials

GROUP CUE

A B C D AC BD

Explicit .970+0.010 .955+0.020 .962+0.017 .964+0.011 .956+0.014 .934+0.032

Partial Exp .944+0.016 .931+0.026 .950+0.017 .937+0.017 .943+0.017 .936+0.018

Implicit .966+0.004 .974+0.010 .957+0.009 .963+0.009 .962+0.011 .952+0.015

Means (+ SEMs) from Experiment 1

Percent Correct on Predict Incompatible Trials

GROUP CUE

A B C D AC BD

Explicit .854+0.039 .909+0.017 .855+0.030 .870+0.024 .825+0.038 .913+0.012

Partial Exp .832+0.039 .894+0.026 .824+0.041 .835+0.037 .829+0.033 .904+0.022

Implicit .826+0.024 .888+0.026 .813+0.031 .833+0.034 .847+0.036 .886+0.024
