# Supplementary material for: Conflict Adaptation and Cue Competition during Learning in an Eriksen Flanker Task
Source: PLoS One. 2016 Dec 12;11(12):e0167119. doi: 10.1371/journal.pone.0167119 (PMC5152815; doi:10.1371/journal.pone.0167119)
Supplement: S3 Table — Percent compatible choices in a probability-estimation task given by the explicit, partial-explicit, and implicit instructed subjects for all the cues. (DOCX) [file pone.0167119.s003.docx]

Table 3: Means (+ SEMs) from Experiment 2

Reaction Time (Noise Compatibility Effect in milliseconds)

GROUP CUE

A B C D AC BD

Explicit 59.46+6.82 39.46+5.34 54.08+7.94 53.23+4.70 56.69+7.15 36.62+5.98

Partial Exp 53.62+7.49 33.46+9.44 47.15+6.80 56.08+7.00 52.77+7.33 27.77+8.53

Implicit 44.62+6.59 45.85+8.43 60.62+6.09 60.15+7.47 51.31+7.10 43.23+5.74

________________________________________________________________________________

Errors (Noise Compatibility Effect: Errors on Inompatible Trials – Errors on Compatible Trials)

GROUP CUE

A B C D AC BD

Explicit 0.11+0.02 0.03+0.01 0.08+0.02 0.09+0.02 0.12+0.02 0.03+0.01

Partial Exp 0.08+0.01 0.05+0.01 0.08+0.01 0.10+0.01 0.07+0.01 0.04+0.01

Implicit 0.06+0.01 0.05+0.01 0.05+0.01 0.06+0.02 0.03+0.01 0.01+0.01
