# Supplementary material for: Conflict Adaptation and Cue Competition during Learning in an Eriksen Flanker Task
Source: PLoS One. 2016 Dec 12;11(12):e0167119. doi: 10.1371/journal.pone.0167119 (PMC5152815; doi:10.1371/journal.pone.0167119)
Supplement: S4 Table — Means (+ SEMs) from Experiment 1 (DOCX) [file pone.0167119.s004.docx]

Table 4: Means (+ SEMs) from Experiment 2

Reaction Time on Predict Compatible Trials

GROUP CUE

A B C D AC BD

Explicit 452.1+22.6 452.2+22.8 456.8+21.5 450.5+21.5 448.8+22.7 450.6+21.0

Partial Exp 444.2+13.7 452.4+13.4 445.9+15.5 445.7+13.7 444.2+14.7 449.5+16.0

Implicit 479.9+13.0 485.3+14.7 477.5+12.3 476.5+12.1 480.2+12.9 477.4+12.5

________________________________________________________________________________

Reaction Time on Predict Incompatible Trials

GROUP CUE

A B C D AC BD

Explicit 511.5+25.8 491.7+25.8 511.0+26.4 503.7+24.8 505.5+26.6 487.2+24.5

Partial Exp 497.8+16.0 485.8+17.4 493.1+16.2 501.8+16.8 496.9+15.9 477.3+16.4

Implicit 524.5+14.2 531.2+15.5 538.1+14.1 536.6+13.9 531.5+14.1 520.6+14.7

Means (+ SEMs) from Experiment 2

Percent Correct on Predict Compatible Trials

GROUP CUE

A B C D AC BD

Explicit .970+0.005 .959+0.013 .975+0.004 .971+0.005 .969+0.003 .954+0.010

Partial Exp .957+0.011 .968+0.012 .964+0.009 .958+0.008 .963+0.011 .958+0.011

Implicit .960+0.010 .978+0.007 .966+0.007 .957+0.010 .963+0.007 .950+0.010

Percent Correct on Predict Incompatible Trials

GROUP CUE

A B C D AC BD

Explicit .861+0.022 .930+0.007 .895+0.016 .881+0.016 .854+0.022 .931+0.007

Partial Exp .878+0.021 .920+0.009 .880+0.016 .863+0.016 .894+0.016 .922+0.008

Implicit .904+0.008 .928+0.009 .912+0.013 .895+0.013 .928+0.009 .939+0.008
