# Supplementary material for: Conflict Adaptation and Cue Competition during Learning in an Eriksen Flanker Task
Source: PLoS One. 2016 Dec 12;11(12):e0167119. doi: 10.1371/journal.pone.0167119 (PMC5152815; doi:10.1371/journal.pone.0167119)
Supplement: S1 File — Ethics Statement. (DOCX) [file pone.0167119.s005.docx]

Ethic Statement

This research project was approved by the University of Missouri Campus Institutional Review Board (Approval Number 1071138).
